# Community based weighing of newborns and use of mobile phones by village elders in rural settings in Kenya: a decentralised approach to health care provision

**DOI:** 10.1186/1471-2393-12-15

**Published:** 2012-03-19

**Authors:** Peter Gisore, Evelyn Shipala, Kevin Otieno, Betsy Rono, Irene Marete, Constance Tenge, Hillary Mabeya, Sherri Bucher, Janet Moore, Edward Liechty, Fabian Esamai

**Affiliations:** 1 Department of Child Health, Moi University School of Medicine, Eldoret, Kenya; 2 Moi Teaching and Referral Hospital, Eldoret, Kenya; 3 Department of Pediatrics, Indiana University School of Medicine, Riley R208 699 Riley Hospital Drive, Indianapolis, IN, USA; 4 RTI International, Research Triangle Park, NC, USA

**Keywords:** Village elders, Birth registry, Community health workers

## Abstract

**Background:**

Identifying every pregnancy, regardless of home or health facility delivery, is crucial to accurately estimating maternal and neonatal mortality. Furthermore, obtaining birth weights and other anthropometric measurements in rural settings in resource limited countries is a difficult challenge. Unfortunately for the majority of infants born outside of a health care facility, pregnancies are often not recorded and birth weights are not accurately known. Data from the initial 6 months of the Maternal and Neonatal Health (MNH) Registry Study of the Global Network for Women and Children's Health study area in Kenya revealed that up to 70% of newborns did not have exact weights measured and recorded by the end of the first week of life; nearly all of these infants were born outside health facilities.

**Methods:**

To more completely obtain accurate birth weights for all infants, regardless of delivery site, village elders were engaged to assist in case finding for pregnancies and births. All elders were provided with weighing scales and mobile phones as tools to assist in subject enrollment and data recording. Subjects were instructed to bring the newborn infant to the home of the elder as soon as possible after birth for weight measurement.

The proportion of pregnancies identified before delivery and the proportion of births with weights measured were compared before and after provision of weighing scales and mobile phones to village elders. Primary outcomes were the percent of infants with a measured birth weight (recorded within 7 days of birth) and the percent of women enrolled before delivery.

**Results:**

The recorded birth weight increased from 43 ± 5.7% to 97 ± 1.1. The birth weight distributions between infants born and weighed in a health facility and those born at home and weighed by village elders were similar. In addition, a significant increase in the percent of subjects enrolled before delivery was found.

**Conclusions:**

Pregnancy case finding and acquisition of birth weight information can be successfully shifted to the community level.

## Background

A major challenge for health care systems in developing countries is access to accurate vital statistics data. This is particularly problematic where significant proportions of health care are delivered outside of the health care system. In sub-Saharan Africa, much obstetric care is provided in the village by traditional birth attendants. Hence, the outcomes of these pregnancies are not included in the vital statistics registers of the health and demographic system. Identifying pregnancies and obtaining birth weights in the village, while not technically difficult, would be time consuming and expensive if done by health care professionals. These tasks might therefore be ideal for shifting to lay personnel.

An evidence base is required to document that health care tasks can be safely shifted from high level health care professionals to lower level workers or lay persons at the community level in low resource settings [1,2]. Where needed, this may include creating new cadres of community workers based on the local context [1,2]. The Douala Plan of Action, which was adopted at the Conference on Human Resources for Health in June 2007, articulates the need to train community-based health workers according to the country's needs [3]. One task that lay leaders in rural areas might assume is obtaining vital statistics data, including registering births and obtaining birth weights.

The Maternal and Neonatal Health (MNH) Registry Study is a multicenter study in seven countries worldwide, including Kenya. It seeks to register all births and neonatal and maternal deaths, regardless of whether the delivery occurred within a health facility or within the home. These data then become available to the health care system, and also provide the baseline data for other intervention studies aimed to improve perinatal/neonatal outcomes. These interventions include the Global Network Emergency Obstetric and Neonatal Care (EmONC) study [1]. Birth weights of all newborns therefore need to be obtained and documented [2,4]. In the initial phase of the study, actual rather than estimated newborn weights had been obtained in only 30% of Kenyan births, nearly all from those infants born in health facilities where weighing scales are available. The analysis of the data showed most weights were missing for those delivered at home. Thus, a system was devised to solve the challenge of obtaining these weights in the community.

The lowest administrative level defined by the Government of Kenya (GOK) is the village. Each village has between 10-50 households. The GOK administrator in a village is a Village Elder. This is an official position within the Kenyan government, elected by the people of the village. One of the village elder's responsibilities is to record home births and deaths that have occurred. The study team decided to leverage this function, by asking the elders to also obtain birth weights. To assist them in this task, each elder was provided a platform balance weighing scale and a mobile phone.

This paper describes a comparison of the newborn-weight and pregnancy case finding data obtained before and after provision of weighing scales and mobile phones to village elders. It also discusses the issues raised by the use of village elders in obtaining health information.

## Methods

The Global Network for Women's and Children's Health Birth Registry is a prospective observational study of pregnancy outcomes in six countries (Kenya, Zambia, India, Pakistan, Guatemala, and Argentina). The methods and initial results have been described in detail [3]. In brief, this is a non-intervention study, and its goal is to generate data to accurately estimate perinatal, neonatal, and maternal mortality in these countries. To do so, the study attempts to register and follow to 42 day outcome every pregnancy that occurs within the geographic boundaries of the study clusters.

For the purposes of this paper, as well as the overall Registry study, enrollment signifies the informed consent of the pregnant women for data collection and analysis of her pre and postnatal data. Enrollment does not imply any particular participation by her in the health care delivery system, although enrolled women were encouraged to seek prenatal care. More than 99% of women approached for enrollment consented.

This was a six-month pre- and post-analysis of changes in several process indicators of the Registry after providing mobile phones and weighing scales to village elders. Sixteen geographically distinct clusters were formed using level 6 administrative boundaries, called locations. All clusters were within Western Province. Provinces in Kenya are divided into smaller administrative units, called Districts. The study areas covered the districts of Teso North, Teso South, Bungoma East, Bungoma West, Bungoma South, Mumias, Butula, Nambale and Busia Districts. Each district has Divisions which are further subdivided into Locations, each headed by an elected Chief. Each location has sub-locations and villages, each village headed by an administrator, the Village Elder. There is bidirectional reporting and communication between chiefs and elders. The total number of villages involved was 474.

Weighing scales and mobile phones were provided to the 474 village elders between July and September, 2009. Elders were initially trained in a group setting with all elders from a given cluster, with use of phones and weighing scales demonstrated by the study coordinators. They were subsequently given one-on-one training by the cluster registry administrator until mastery of all aspects of their task was demonstrated. This included accurately weighing infants, recording the weights, and using the phone to contact the registry administrator. Ongoing feedback was provided to the elders by the registry administrators. Data collected in the six months prior to July (i.e. January 1 - June 30, 2009) were used for the pre-distribution phase statistics. To assure that all scales and phones were distributed and in use, the post-distribution phase statistics comprised data collected November 1 2009 through April 30, 2010. Data collection has continued after the completion of the post-distribution six months. These follow up data are presented for May through December, 2010.

**Figure 1:**
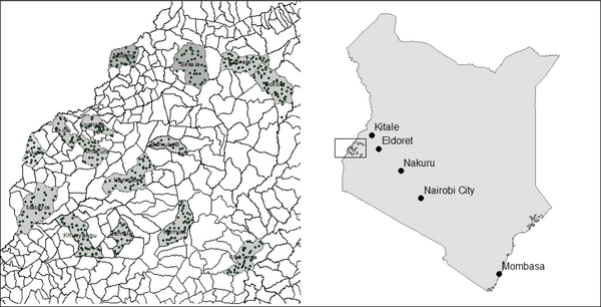
**Location of the Global Network - Kenya site**. The left panel depicts each of the 16 clusters. Each dot represents an individual village headed by a village elder. The right panel shows where within Kenya the clusters are located.

The exact location of each elder's home was determined by a hand held GPS unit and marked on the map in Figure 1. This region of western Kenya is highly rural and has a high incidence of poverty. Most families rely upon subsistence farming. Each cluster is served by one government health facility capable of conducting deliveries, as well as several lower level health facilities or dispensaries. In addition there are a few mission hospitals also providing delivery service. This area has approximately 90% mobile telephone network coverage.

Data were collected in each cluster by a registry administrator, employed by the investigators. This person was responsible for identifying all pregnancies and deliveries within the geographic bounds of the cluster, obtaining informed consent for data collection, completing the data entry forms for the study, and passing the completed data forms on to the investigators. Birth weight and pregnancy outcome data for birth occurring within a health facility were taken directly from facility records and recorded onto study data forms by the registry administrator.

In our population, 90% of women enrolled in this study report attending least 1 antenatal care clinics. Enrollment in the study was a separate process from ANC attendance. Some women were in fact enrolled at the time of ANC, but many were enrolled in the village at a time distinct from ANC. This was due to the fact that each cluster had a single Registry Administrator responsible for enrolling subjects, whereas ANC clinics were held simultaneously in multiple facilities within each cluster. For the purposes of the present report, enrollment refers to the woman granting permission to the registry administrator for data collection.

The weighing scale and mobile phone distribution phase involved the District Chiefs who invited all village elders to their offices. The study team of the MNH Registry brought the phones and weighing scales to the Chiefs' meetings. The type of phone was Nokia 1202 (Nokia, Keilalahdentie 2-4, Finland). The phones could send and receive short text messages and voice calls. All village elders were asked to purchase airtime credit for the mobile phone as it was not provided by the study. The weighing scale was Model RGZ -20 (Wuxi Weight Factory, Wuxi City, China). This is a simple, mechanical platform scale with precision to the nearest 50 grams. The MNH study staff trained the chiefs and village elders on how to use the phones and weighing scales, including how they were to be operated, maintained and stored. Elders were also provided with a notebook and a pen to record measurements. Each village elder and her/his respective registry administrator determined individually how they would communicate with one another for reporting of pregnancies and birth weights.

Additional instructions included an understanding that the phones were the property of the study and that the elder would be expected to replace lost phones. They were asked to ensure that all infants were weighed immediately at birth or as soon as possible, but no later than one week post-delivery.

Discussions were conducted on the most suitable approaches to weighing infants born at home in each village. These included either the babies being taken to the village elders' home or the weighing scale being taken by the elder to the household where the birth had occurred. In all cases the decision was to maintain the scale in the elder's home and have the mother bring the infant to the elder for weighing. The chiefs agreed to provide supportive supervision for weighing of these newborns and communication of the data to the Registry Administrators.

This is a population based study of all perinatal mortality within the study geographic confines; therefore, no predetermined sample size was estimated.

Ethical approval for this study was granted by the Institutional Research Ethics Committee of Moi University and by the Institutional Review Board for the Protection of Human Subjects of Indiana University and RTI International.

The primary outcome of the study was the change in percentage of measured birth weights in the post-distribution phase. A secondary outcome was the change in percentage of women enrolled before the delivery of the infant, a surrogate for improved case finding after the intervention. In addition, we also examined the distribution of birth weights measured in a health facility compared to those measured in the village elder homes. This comparison was done only for infants born in the post distribution phase. Data are presented as aggregate numbers and percentages or as monthly percentage. Statistical significance was determined by *t*-test.

## Results

**Figure 2:**
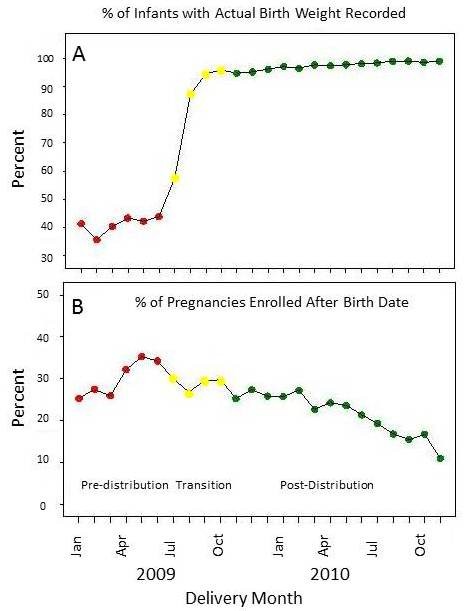
**Top panel shows the percent of infants in each month that had an actual birth weight recorded**. The lower panel shows the percent of pregnancies in each month enrolled after the birth date.

The total number of births in the period between January 2009 and December 2010 was 17,115 (Table 1). Figure 2A shows the percentage of exact weights missing from the Data Management System during this time period. Only weights taken and recorded within 7 days of the birth were considered accurate birth weights. The percentage of exact weight recorded ranged from less than 40% before the intervention to nearly 100% after the intervention.

**Table 1:** Babies weighed and late enrollment by distribution time period

	Jan 09-Jun 09	Jul 09-Oct 09	Nov 09-Apr 10	May 10-Dec 10	Total
Infants delivered, N	3,895	3,087	4,197	5,936	17,115
Birth weight measured, N (%)	3,894	3,087	4,197	5,936	17,114
Yes	1,615 (41.5)	2,609 (84.5)	4,040 (96.3)*	5,844 (98.5)	14,108 (82.4)
No	2,279 (58.5)	478 (15.5)	157 (3.7)	92 (1.5)	3,006 (17.6)
Birth weight measured within 7 days, N (%)	1,615	2,609	4,040	5,844	14,108
Yes	474 (29.3)	1,868 (71.6)	3,579 (88.6)	5,480 (93.8)	11,401 (80.8)
No	1,141 (70.7)	741 (28.4)	461 (11.4)	364 (6.2)	2,707 (19.2)
Enrolled after delivery, N (%)	3,891	3,081	4,194	5,919	17,085
Yes	1,183 (30.4)	893 (29.0)	1,076 (25.7)*	1,104 (18.7)	4,256 (24.9)
No	2,708 (69.6)	2,188 (71.0)	3,118 (74.3)	4,815 (81.3)	12,829 (75.1)
Birth weight > 5500 g for babies measured within 7 days, N (%)	474	1,868	3,579	5,480	11,401
Yes	0 (0.0)	0 (0.0)	0 (0.0)	1 (0.0)	1 (0.0)
No	474 (100.0)	1,868 (100.0)	3,579 (100.0)	5,479 (100.0)	11,400 (100.0)
Birth weight > 5500 g, N (%)	1,615	2,609	4,040	5,844	14,108
Yes	6 (0.4)	22 (0.8)	8 (0.2)	6 (0.1)	42 (0.3)
No	1,609 (99.6)	2,587 (99.2)	4,032 (99.8)	5,838 (99.9)	14,066 (99.7)
Facility Birth weight > 5500 g, N (%)	1,353	976	1,333	2,158	5,820
Yes	1 (0.1)	3 (0.3)	1 (0.1)	1 (0.0)	6 (0.1)
No	1,352 (99.9)	973 (99.7)	1,332 (99.9)	2,157 (100.0)	5,814 (99.9)
Home Birth weight > 5500 g, N (%)	262	1,632	2,707	3,683	8,284
Yes	5 (1.9)	19 (1.2)	7 (0.3)	5 (0.1)	36 (0.4)
No	257 (98.1)	1,613 (98.8)	2,700 (99.7)	3,678 (99.9)	8,248 (99.6)

*- Post-distribution differs from pre-distribution, *p *< 0.0001

Of the 17,115 total births, still births accounted for **324 **and early neonatal deaths (< 7 days) accounted for **208**; 40% of still births and 61% of early neonatal deaths were weighed. Obtaining accurate birth weights for home delivered still births and early neonatal deaths continues to be difficult Cultural practices in this context dictate prompt burial of still borns and infant who have died, with burial taking place before either the village elder or registry administrator were aware of the birth.

An additional benefit of the project was earlier pregnancy case discovery and subject enrollment. This is demonstrated in Figure 2B. Before the introduction of the scales and mobile telephones, nearly 40% of subjects were enrolled after delivery. Comparing the 6 months prior to six months after provision of mobile phone and weighing scales to village elders, the subjects identified after delivery declined from an average of 30.4% pre-distribution to 25% post-distribution (*p *< 0.0001). This percentage has continued to decline during the follow-up phase, and was 12% for December 2010.

**Figure 3:**
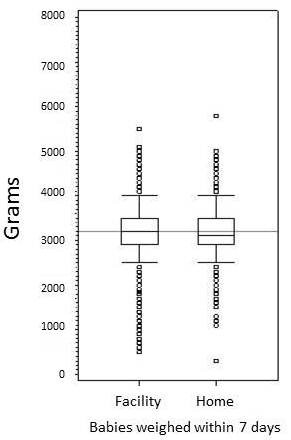
**Box-plot of birth weight distributions for infants born and weighed in a health facility and those born at home and weighed by a village elder**. The box encloses 25^th ^to 75^th ^percentile, and whiskers denote 1st quartile - 1.5(interquartile range) and 3 rd quartile + 1.5(interquartile range). Medians were compared by Wilcoxon test.

The birth weight distributions in the post distribution phase for infants born at home and weighed by elders were compared to those born in a health facility and weighed by a health care professional. Figure 3 shows box plots for the two cohorts, demonstrating the numbers and weights of the outliers in each group. A slightly higher percentage of birth weights obtained by elders were non-plausible, defined as > 5.5 kg in the home birth cohort (17/6171, 0.27%) than in the health facility birth cohort (5/3288, 0.15%). When only infants weighed within 7 days of birth were included in the analysis (Figure 3), there were no infants found to have a birth weight greater than 5500 g.

After the initial capital expense, costs for maintenance and replacement have not been excessive. Over a 12 month period three (3) mobile phones (0.6%) have been lost, one (1) could not be traced when a village elder died and two (2) were not returned when the village elders left their positions. Fifteen (15) weighing scales (3%) have needed to be repaired.

## Discussion

The introduction of mobile phones and weighing scales to the community through 474 village elders in 16 study clusters was followed by an immediate and significant decrease of the number of newborns without exact birth weights. The proportion of hospital delivery remained the same during the period of this study. This suggests that it was possible to involve the lowest administrative heads at the community level to gather vital statistical data, that mobile phones may have helped to link community workers to a data management system, and that the village elders can be effective in introducing specific interventions quickly to a community.

Other investigators have introduced devices for weighing newborns in field settings. Most of these devices have been low cost sling type scales, such as those developed by PATH. We elected to use a platform scale for several reasons. First of all, we desired greater precision than the sling scales are capable of providing. The initial version of the PATH scale only discriminated between LBW < 2500 g versus normal weight > 2500 g. A second version allowed for color coded increments of 500 g (> or = 2500 g, 2000-2499 g, < 2000 g) [5]. The scale we chose is precise to 50 g increments and capable of obtaining the full range of expected birth weights. Second, for the sling scale to be accurate it must be held motionless for a long enough period of time for the measurement to stabilize and be recorded. We had concerns about the ability of unsupervised village elders to reliably perform this maneuver. Finally, we had concerns about whether the sling scale would be culturally acceptable in the rural Kenyan setting.

The birth weight distribution was similar in the home birth cohort compared to the cohort born in a health facility. Although the birth weight distributions were similar, there were more very low birth weight (VLBW) infants in the facility birth cohort and more infants greater than 4.0 kg in the home delivery cohort. This could be the result of a few more women in premature labor self-selecting to deliver in a health facility, accounting for the greater number of infants with very low birth weight in the facility group. It could also be the result of infants in the home birth cohort being weighed at greater than 7 days of age and beginning postnatal weight gain. Alternatively, it could be the result of small systematic errors in the weighing of the home birth cohort. Nevertheless, the similarity of birth weight distributions gives credence to the hypothesis that village elders, even if non literate, can be relied upon to obtain, record, and communicate reasonably accurate birth weights.

In other studies mobile phones have been used for communicating health messages [6-9] and for gathering data [6,8,10,11]. The present data suggest successful communication of birth weight data between the community and a vital statistics data management system is feasible. It would be reasonable to suggest that the mobile phone was the major factor that enhanced communication between the village elder and the registry administrator. However, other factors such as involvement of the chief and provision of the weighing scales may also have played a role. Process related information was not obtained for this study and thus we could not make conclusive inference. Clearly, close cooperation and open lines of communications between the village elder and the data transcriber within the health facility are necessary for this system to function well.

Village elders know all household members in their village. They assist the local administration in maintaining civil order. They perform roles delegated to them by the chiefs. This includes gathering information from the village and at times conveying messages from the chief to the villages. They also routinely gather data about home deliveries and deaths in the villages. This was a novel study involving the village level administrators to gather demographic data. These results infer exciting possibilities related to creation of new cadres of health workers, training, standards, and distribution of tasks in the community.

Of importance to our study was the finding that engagement of village elders enhanced pregnancy case discovery by the registry administrator, with the result that more pregnancies were discovered and enrolled before delivery. These women represent the "hard to reach" fraction of pregnancies, women whose statistics often are missing from birth registry data. Failure to enroll these women in a timely manner likely results in underestimation of both maternal and perinatal/neonatal mortality rates.

Information on new cadres of community health workers and the resulting WHO recommendation on Task-Shifting has mainly been from home-based care of HIV positive patients [12]. Community Health Workers as outlined in the Kenya Ministry of Health Strategic Plan are assumed to be either retired nurses, women interested in health matters or Traditional Birth Attendants [13] This result seems to suggest that village elders, many of whom are older males, can also be used to solve the challenge of obtaining accurate demographic information in the community. It will be important that such persons, trained within the context of a specific project, be retained by country Ministries of Health or of Demographic Services at the conclusion of the specific project. This can be encouraged by data sharing between the project and the Ministry, as was done in the current project. Village elders reported data not only to study personnel but also to the village chiefs, local elected officials charged with the task of collecting village level vital statistics. It is hoped that this will discourage future projects from establishing parallel and redundant data collection systems, but rather strengthen the capacity of the local government to collect complete and accurate demographic data.

The quality assurance of the tasks done by health workers in the community has been of great interest [14-18]. It is outside the scope of this paper to discuss the accuracy of the data obtained from all areas in the study. Village elders may require more intensive training compared to other cadres that exist outside the formal health ministry structure. On the other hand, anecdotal observations suggest that they may be having more free time than other cadres in the community, which would potentially enable them to take up some health system tasks.

## Conclusions

We conclude that introduction of weighing scales and mobile phones through village elders improved the number of weighed infants. There is an unexploited potential in village elders doing some basic health related tasks in the community setting that needs further exploration.

We recommend the use of weighing scales and mobile phones in the community to obtain demographic data in low resource settings with high mobile network coverage. We also suggest the exploration of the village elder as a novel cadre of community health workers.

## Competing interests

The authors declare that they have no competing interests.

## Authors' contributions

PG was the primary author of the manuscript. ES was the study manager and coordinated all logistics for scale and mobile handset distribution. KO was the data manager, supervised all data entry, and collected all GPS data. BR was the direct supervisor of all registry administrators and coordinated their work in the field. IM and CT were each responsible for 8 of the sixteen clusters, conducted community entry meetings, and provided weekly oversight and feedback to registry administrators for the accuracy all data collected. HM assisted IM and CT in community entry meetings, and substituted when they were unavailable. SB was responsible for the preparation of ethics review documents. JM conducted the statistical analysis and prepared the data figures and tables. EL is principal investigator for the overall Global Network project and conducted initial statistical and GPS analysis. FE is co-Principal Investigator of the overall Global Network project and conceived the idea of distributing scales to village elders. All authors read and approved the final manuscript.

## Pre-publication history

The pre-publication history for this paper can be accessed here:

http://www.biomedcentral.com/1471-2393/12/15/prepub
